# Reconfigurable Logic-in-Memory Oxide Transistors Enabled by Transferable Ferroelectric HZO

**DOI:** 10.1021/acsnano.6c04397

**Published:** 2026-06-30

**Authors:** Chang-Chang Huang, Bo-Cia Chen, Hao-Tse Lee, Rahul, Yung-Chi Su, Chien-Ting Wu, Chien-Chung Hsu, Yen-Lin Huang, Jan-Chi Yang, Der-Hsien Lien

**Affiliations:** † Institute of Electronics, 34914 National Yang Ming Chiao Tung University, Hsinchu 300093,Taiwan; ‡ Department of Physics, 34912 National Cheng Kung University, Tainan 701401,Taiwan; § Center for Quantum Frontiers of Research & Technology (QFort), 34912 National Cheng Kung University, Tainan 701401,Taiwan; ∥ Department of Materials Science and Engineering, 34914 National Yang Ming Chiao Tung University, Hsinchu 300093,Taiwan; ⊥ Taiwan Semiconductor Research Institute (TSRI), Hsinchu 300091,Taiwan

**Keywords:** transferable ferroelectrics, HZO, high-κ oxide transistors, logic-in-memory computation, large-area transfer

## Abstract

Oxide semiconductors such as indium oxide (In_2_O_3_) offer high-electron mobility and low-temperature processability, making them promising candidates for back-end-of-line (BEOL)-compatible logic-in-memory applications. However, direct deposition of high-κ ferroelectric dielectrics (Hf_0.5_Zr_0.5_O_2_; HZO) on oxide channels typically degrades interfacial quality, leading to threshold voltage shifts and unstable polarization due to depolarization fields and defect states. In this work, we leverage advanced membrane transfer techniques to demonstrate a transferable ferroelectric HZO layer for interface-layer-free integration with In_2_O_3_. This approach forms a van der Waals-like junction, evidenced by an ∼0.8 nm interfacial gap, which avoids the chemical incompatibilities of conventional gate stack processing while preserving the pristine stoichiometry of the In_2_O_3_ channel. The transferred HZO exhibits a dielectric constant of 26 and low leakage current (<10^–7^ A cm^–2^ at 1 MV cm^–1^) while maintaining robust ferroelectric switching. Dual-gate ferroelectric In_2_O_3_ transistors achieve a large memory window and stable endurance over 10^9^ cycles. We further integrate these devices into reconfigurable inverter circuits that dynamically switch between NOR and NAND logic functions with tunable voltage transfer characteristics. The ferroelectric thin-film transfer process is fully compatible with silicon back-end-of-line thermal budgets and scalable to wafer-level integration, offering a viable route toward high-density, multifunctional logic-in-memory architectures.

## Graphical abstract

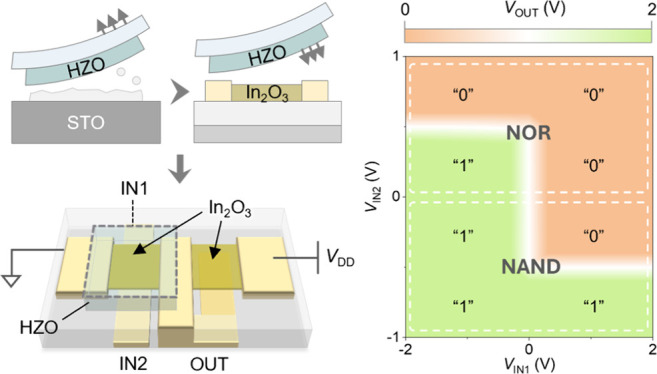

## Introduction

Ferroelectrics based on hafnium zirconium oxide (Hf_0.5_Zr_0.5_O_2_; HZO) have attracted considerable attention for their CMOS compatibility and ferroelectric properties, enabling applications from nonvolatile memory to steep-slope logic devices.^1–15^ These properties are attributed to a metastable orthorhombic phase (o-phase) of HZO, responsible for its switchable polarization behavior.^12,14,16^ However, o-phase formation is thermodynamically disfavored under standard crystallization, as HZO preferentially stabilizes in the nonferroelectric monoclinic or tetragonal phases. This leads to a major challenge for stabilizing the o-phase of HZO. To promote the formation of the ferroelectric o-phase, an interfacial dielectric layer is usually required. ZrO_2_ or Al_2_O_3_ are commonly introduced as interfacial layers to promote the formation of o-phase by providing interfacial lattice constraints.^17–24^ However, the interfacial layers also induce adverse effects, including depolarization field due to incomplete screening of polarization charges and the formation of interfacial trap states, both of which reduce remanent polarization and degrade electronic performance.^25,26^

To address this challenge, eliminating interfacial layers is a promising approach to fully realize the intrinsic ferroelectricity of HZO. Recent advancements show that optimizing deposition conditions can effectively suppress interfacial reactions while preserving HZO ferroelectricity. For example, precise control over the growth temperature or postdeposition annealing promotes o-phase and preserves ferroelectric characteristics.^24,27^ In addition to optimizing HZO, engineering the underlying semiconductor has proven effective in promoting o-phase formation. Highly doped epitaxial GeSi has been demonstrated to screen polarization charge and reduce interfacial defect formation, enabling reliable switching without an interfacial layer.^28^ Oxide materials such as TiO_2_ are chemically compatible with HZO, enabling direct o-phase crystallization without interfacial buffers.^29–31^ However, interfacial-layer-free integration remains challenging due to tight process tolerances and potential long-term interface instability.

Another critical challenge is that even with o-phase HZO, robust ferroelectric functionality is not always achieved, often constrained by the intrinsic properties of the underlying semiconductor channel. Insufficient screening of polarization charges or high interface trap densities can suppress domain formation and polarization switching. For example, 2D transition metal dichalcogenides offer atomically sharp interfaces and low defect densities, but their inert surfaces hinder direct HZO growth, often requiring seed layers or surface treatments.^4,32–35^ Indium oxide (In_2_O_3_) has therefore emerged as a promising alternative, exhibiting high mobility even at 1 nm thickness.^36–41,62–64^ While ferroelectric behavior has been demonstrated in In_2_O_3_/HZO structures, postannealing and dielectric interlayers are often required.^42–47^ These additions can lead to memory window degradation due to insufficient charge compensation, reaffirming that achieving interface-free ferroelectric integration remains a key challenge.

In this work, we demonstrate a transferable ferroelectric HZO layer that enables interface-layer-free integration with In_2_O_3_ semiconductors. The transfer of freestanding oxide membranes using sacrificial LSMO has been recently pioneered for various materials and systems, establishing a foundation for enabling high-performance electronics.^4,13,48–51^ The transferred HZO forms a van der Waals (vdW)-like junction with In_2_O_3_, directly evidenced by an ∼ 0.8 nm interfacial gap, yielding an atomically sharp interface without the need for epitaxy or dielectric buffers. In_2_O_3_ further provides favorable charge balance conditions to support full polarization switching, resulting in a large memory window of 2.3 V. This enhanced performance is attributed to the absence of interfacial layers and the availability of oxygen vacancies that stabilize switching dynamics. Based on the high-quality, transferred ferroelectric HZO, we demonstrate a dual-gate ferroelectric inverter that integrates memory and logic functionality in a single device, enabling tunable voltage-transfer characteristics and reconfigurable inverters for switchable NOR and NAND logic operations. The transfer process is compatible with large-area fabrication and yields statistically reproducible electrical performance across device arrays, establishing a scalable platform for interface-engineered ferroelectric electronics with enhanced multifunctionality.

## Results and Discussion

### Transferable HZO

Figure 1 a schematically illustrates the preparation of transferable ferroelectric HZO thin films, and its applications as a high-κ dielectric on oxide transistors. A thin La_0.7_Sr_0.3_MnO_3_ (LSMO) layer (∼ 20 nm) was epitaxially deposited onto SrTiO_3_ (STO) substrates via pulsed laser deposition (PLD), followed by the growth of an HZO film at 700 °C. The high-temperature growth of HZO facilitates the transition from its thermodynamically stable monoclinic phase to the metastable o-phase, while the subsequent cooling process preserves this ferroelectric structure by suppressing reversion to the monoclinic phase.^12–14^ Here, LSMO is employed as an interfacial layer, leveraging interface termination engineering to enhance HZO crystallization. In addition, LSMO serves as a sacrificial release layer that can be selectively etched using a previously developed wet etching technique, enabling clean separation of the HZO film from the underlying STO substrate. The detached HZO membrane was then transferred onto the target substrate (details in Methods). Note that while thinner films (∼8 nm) can achieve higher remanent polarization via surface energy stabilization (Figure S1), the 20 nm thickness provides better phase stability and lower gate leakage, as has been discussed in our previous study.^4^

**Figure 1:**
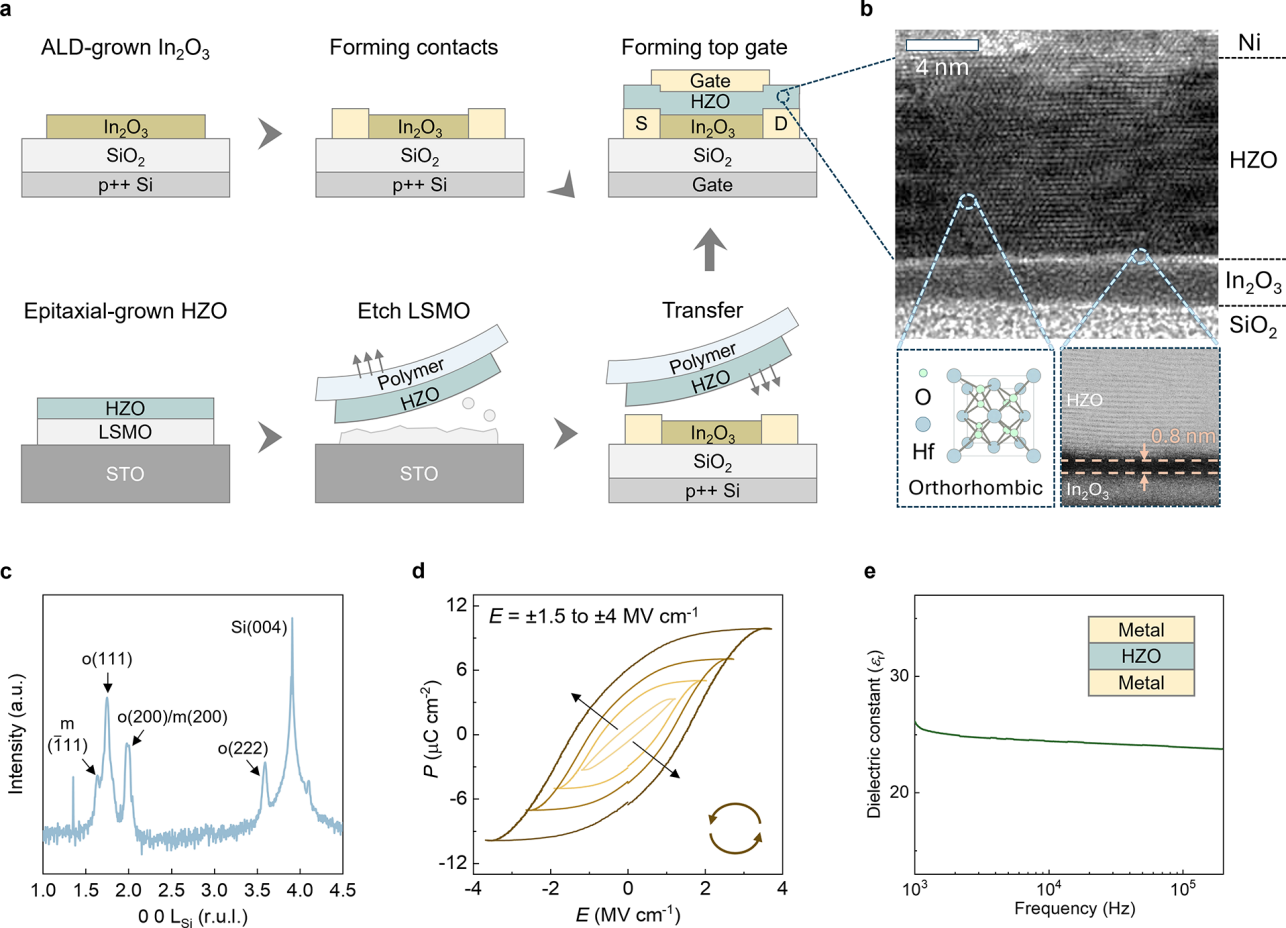
Characterization of our transferable HZO thin film. (a) The synthesis of transferable HZO and fabrication process flow of the transferred-HZO based dual-gated ferroelectric transistors. In_2_O_3_ transistors are fabricated before transfer. (b) HRTEM image of the Ni/HZO/In_2_O_3_/SiO_2_/p^+2^ Si gate stack. HRTEM shows clear crystallization of the transferred HZO of thickness 20 nm and an amorphous 2 nm In_2_O_3_ channel. The inset shows a cross-sectional HAADF-STEM image of the HZO/In_2_O_3_ interface, revealing an ∼0.8 nm interfacial gap consistent with a physically contacted, vdW-like junction. (c) XRD θ-2θ scan for the transferred 20 nm-thick HZO thin film on a Si substrate. (d) Polarization-electric field loops of the metal–insulator–metal structure, where the thickness of HZO is 20 nm. (e) Frequency-dependent dielectric response of HZO thin film at a thickness of 20 nm.

To construct the transistor stack, 2 nm In_2_O_3_ was deposited by atomic layer deposition (ALD) at a temperature of 300 °C on a 50 nm SiO2 on p^+2^ silicon substrates, where SiO_2_ acts as the dielectric and the p^+2^ silicon acts as the bottom-gate. Source and drain contacts were patterned on the In_2_O_3_ channel layer. The prefabricated HZO membrane was then transferred onto the In_2_O_3_, where it adheres via vdW forces, acting as the ferroelectric layer in the top gate. Together with the bottom SiO_2_ dielectric, this forms a double-gate structure. High-resolution transmission electron microscopy (Figure 1b and Figure S2 for elemental mappings) confirms the high crystalline quality of the HZO film and the integrity of the overall transistor structure. The cross-sectional HAADF-STEM image showing the ∼0.8 nm HZO/In_2_O_3_ interfacial gap is provided as inset of Figure 1b. The transferred HZO flakes typically exhibit lateral dimensions of ∼3–5 mm. High-resolution TEM further reveals an ultrafine multidomain microstructure composed of orthorhombic grains ∼10 nm in size with clearly resolved (111)_o_ lattice fringes, confirming that the ferroelectric o-phase and its multidomain configuration are preserved after the transfer process (Figure S3).

X-ray diffraction (XRD) measurements in Figure 1c further verify the predominant presence of the o-phase in the HZO films, indicating that the ferroelectric phase is well-preserved after the transfer process.^10^ To validate the ferroelectric properties of the transferred HZO films, polarization-electric field (*P*-*E*) measurement is performed using a metal–insulator–metal structure (Figure 1d). Clear hysteresis was observed in the transferred HZO films with the remanent polarization P_r_ of 6.1 μC/cm^2^ and coercive fields of 1.47 MV/cm. The coercive field of our transferred HZO is comparable to other ALD as-grown HZO, potentially resulting in better memory-state distinguishability and resistance to fluctuation.^8,9^ Frequency-dependent capacitance measurements (Figure 1e) reveal that the relative permittivity of the transferred HZO film is approximately 26 and remains stable across a broad frequency range. This stability contrasts with the behavior of many high-κ dielectrics, which often exhibit frequency dispersion due to mechanisms like dielectric relaxation and interface trap responses.^52^ Frequency-dependent C–V measurements (Figure S4) further compare the trap dynamics between the transferred and directly grown HZO. The result shows that the directly grown HZO exhibits pronounced frequency dispersion, where the strong enhancement of capacitance at low frequencies indicates slow-moving charge species, such as interface traps or mobile ionic defects. In contrast, the transferred HZO displays a markedly reduced frequency dispersion with a relatively flat capacitance response across the measured range. This suppression of low-frequency enhancement suggests a substantial reduction in extrinsic charge-screening effects, consistent with a low-defect interface.

### Interfaces of Transferred HZO-In_2_O_3_

Transferable insulating layers, such as hexagonal boron nitride (hBN), have been widely adopted in low-dimensional electronics for their atomically flat surfaces and vdW interfaces, which suppress interface trap densities and reduce charge impurity scattering.^4,53–58^ A similar strategy is applied here with transferable HZO, where the vdW interface not only preserves interfacial quality but also mitigates a critical failure mode in In_2_O_3_-based transistors. While HZO is a strongly ionically bonded oxide, recent studies show the detached membrane can be integrated via a physically transferred, nonchemically bonded interface.^4,13,48–51^ This vdW-like junction is likely enabled by the passivation of surface dangling bonds with hydroxyl groups during the acidic etching process, allowing for integration without forming strong covalent or ionic bonds with the underlying channel. On the other hand, direct deposition of high-κ dielectrics, including HfO_2_, Al_2_O_3_, or HZO, often involves high temperatures or reactive precursors that scavenge oxygen from In_2_O_3_, causing excessive electron doping and large negative threshold voltage (V_T_) shifts that prevent device turn-off (Figure 2a).^38,59^ Transferred HZO avoids this chemical interaction by decoupling high-temperature crystallization from channel integration, thereby eliminating oxygen scavenging and maintaining the pristine electronic properties of In_2_O_3_ (optical image of the transferred device in Figure S5). As a result, devices with transferred HZO exhibit no measurable V_T_ shift (Figure 2a), confirming the vdW-based HZO effectively circumvents the chemical incompatibility inherent to gate stack processing. Transfer characteristics of the ferroelectric In_2_O_3_ transistors, measured by sweeping the top-gate voltage in both polarities (+V_G_^FE^ to -V_G_^FE^ and -V_G_^FE^ to + V_G_^FE^), exhibit a pronounced counterclockwise hysteresis in the I_D_-V_G_^FE^ curves (Figure 2b). This behavior arises from the ferroelectric switching of HZO,^15^ consistent with the *P*–*E* hysteresis observed in Figure 1d.

**Figure 2:**
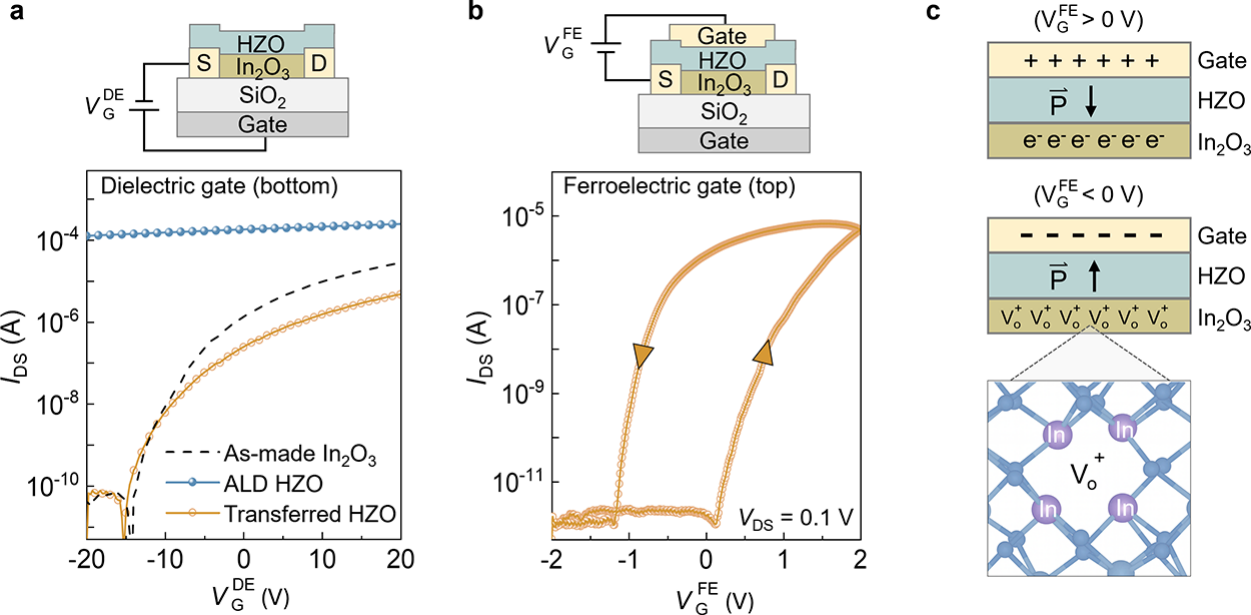
Transfer characteristics of transferred vs ALD-grown HZO/In_2_O_3_ and charge compensation. (a) Bottom-gate transfer curves (I_DS_–V_G_^DE^ curves) for 2 nm-thick In_2_O_3_ devices with different conditions. The case of atomic layer deposition-grown HZO thin film shows a significant V_T_ shift, whereas the case of transferred HZO thin film retains a similar position of V_T_ as in the case of as-made In_2_O_3_ before any top-gate dielectric capping. (b) Top-gate transfer curves (I_DS_–V_G_^FE^ curves) for a 2 nm-thick In_2_O_3_ device. A counterclockwise hysteresis loop is observed due to the ferroelectric polarization switching effect. (c) Conditions for complete polarization switching of our metal/ferroelectric/semiconductor structure based on charge balance condition.

In conventional ferroelectric FETs, an interfacial dielectric is often inserted to form a metal-ferroelectric-insulator-semiconductor (MFIS) structure, where hysteresis arises from polarization switching in the ferroelectric and its electrostatic coupling with the dielectric. The charge balance can be expressed as Q_s_ + Q_de_ + Q_it_ = P_FE_ + ε_0_ε_FE_E_FE_, with Q_s_ and Q_de_ representing charges in the semiconductor channel and dielectric layer, Q_it_ the total interface trap charge (including both the top HZO/In_2_O_3_ and the substrate-side SiO_2_/In_2_O_3_ interfaces), P_FE_ the ferroelectric polarization, and ε_0_ and ε_FE_, and E_FE_ the vacuum permittivity, ferroelectric dielectric constant, and electric field, respectively (derivation in Supporting Information).^25,26,32,46^ Full polarization switching requires both positive and negative compensating charges at the ferroelectric interface; insufficient compensation leads to suppressed hysteresis.

In_2_O_3_ uniquely enables robust ferroelectricity without an interfacial dielectric due to its intrinsic self-compensation. Under negative gate bias, oxygen vacancies act as bound positive charges, while under positive bias, free electrons supply negative charges (Figure 2c). This dual mechanism sustains full polarization switching in transferred-HZO/In_2_O_3_ stacks, avoiding the depolarization and performance loss seen in conventional dielectric-buffered interfaces. To verify this vacancy-driven mechanism, we performed controlled oxygen annealing experiments to modulate the vacancy density (Figure S6). Reducing the oxygen vacancy concentration leads to a significant decrease in the memory window, confirming that ionized oxygen vacancies act as the primary positive charge compensation source required for polarization switching in the absence of thermally generated minority carriers. This self-compensation for the transferred-HZO/In_2_O_3_ stack not only eliminates the need for a dielectric interlayer but also ensures robust ferroelectric hysteresis without the performance degradation commonly associated with interfacial layers.^46^

### Memory Characteristics of Ferroelectric-Gated Oxide Transistors

Building upon the large hysteresis window, the nonvolatile memory functionality of the ferroelectric-gated oxide transistors is examined. The memory window, defined as the threshold voltage difference (ΔV_T_) between forward and reverse top-gate sweeps, exhibits a clear dependence on the maximum applied gate voltage (Figure 3a and Figure S7). Meanwhile, the device exhibits a low leakage current (<10^–7^ A cm^–2^ at 1 MV cm^–1^), benefiting from the combined advantages of robust ferroelectric integrity of HZO and the wide band gap of In_2_O_3_ (>3 eV). As the top-gate voltage increases, the memory window correspondingly expands (Figure 3b), aligning with the voltage-dependent *P*–*E* hysteresis loops shown in Figure 1d. A maximum memory window of 2.3 V is achieved at a sweep range of ± 2.5 V. Pulse-width-dependent measurements show that the memory window remains stable from 10 ms down to 1 μs (Figure 3b), confirming microsecond-scale program/erase operation. This substantial memory window indicates effective polarization switching based on the transferred HZO, without performance degradation from depolarization fields or interfacial charge trapping. In contrast, conventional MFIS stacks often suffer from voltage drops across the interfacial dielectric layer, leading to incomplete polarization switching and reduced memory windows. For instance, HZO-based ferroelectric transistors with interfacial layers have memory windows of around 1.3–1.8 V under similar operating conditions. To facilitate a more direct comparison across different studies, we normalize the memory window by the applied gate voltage, yielding a memory window-to-V_G_^FE^ ratio of 0.46 in our device, which surpasses the ratios reported in previous works (comparison of memory window-to-V_G_^FE^ ratio with other works is shown in Table S1 in Supporting Information).^4,28,29,32–35,42–44,46,47,60,61^ Despite a relatively low P_r_ value of 6.1 μC/cm^2^ and a high coercive field of 1.47 MV/cm compared to previous reports,^9^ the large memory window achieved at low operating voltage underscores the advantage of our transferred HZO integration.

**Figure 3:**
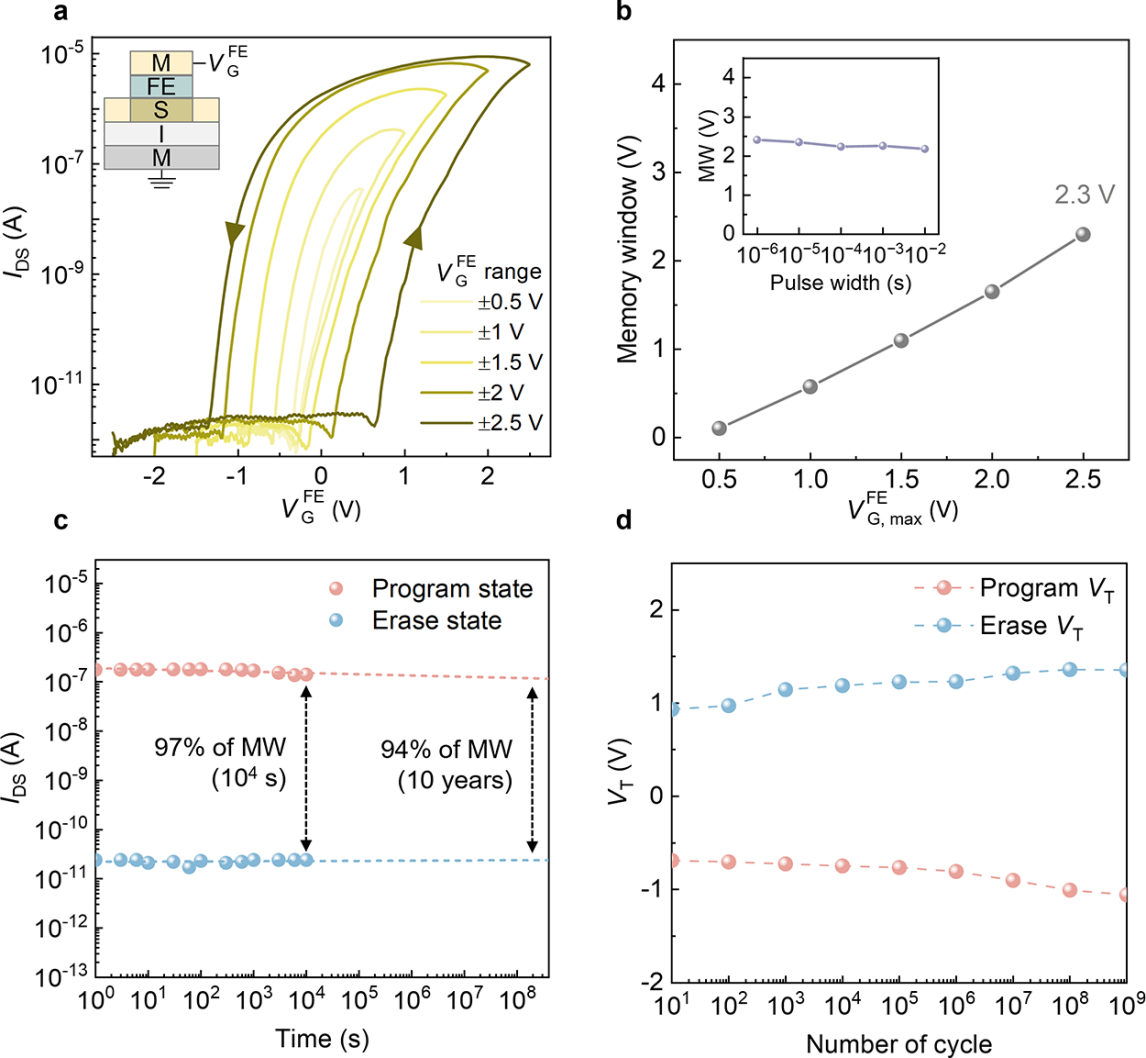
Electrical characteristics and memory behavior of transferred HZO-based ferroelectric transistors with ultrathin In_2_O_3_. (a) I_DS_–V_G_^FE^ curves for a 2 nm-thick In_2_O_3_ ferroelectric transistor. A stable counterclockwise hysteresis with a memory window up to 2.3 V is available. The device has a channel width of 10 μm and a channel length of 3 μm, with a drain-to-source voltage V_DS_ = 0.1 V. (b) Memory window versus V_G_^FE^ of the In_2_O_3_ device. The maximum memory window is 2.3 V at V_G_^FE^ = 2.5 V. The inset shows the pulse-width-dependent memory window, which remains stable from 10 ms down to 1 μs, confirming microsecond-scale program/erase operation. (c) Retention characterization of the In_2_O_3_ device at V_DS_ = 0.1 V, with drain current measured at V_G_^FE^ = 0.5 V and V_G_^DE^ = 0 V. The initial program/erase pulse amplitude is +3 V/-3 V with a pulse width of 50 ms. By extrapolating the decay of the memory window to 10^8^ s (∼10 years, the industry standard for evaluating nonvolatile memory retention), the window retains approximately 94% of its original value. (d) Endurance property for the In_2_O_3_ device at V_DS_ = 0.1 V. The repeating pulse of +3 V/-3 V with pulse width of 1 μs is applied for the endurance test.

Reflecting the large memory window, our device demonstrates an on/off current ratio exceeding 4 orders of magnitude, which remains stable (∼10^4^) over a 10,000 s retention test (Figure 3c). Extrapolating the memory window decay to 10^8^ s (∼10 years) indicates a 94% retention of the original value, confirming that the metastable o-phase remains robust against relaxation into the nonferroelectric m-phase under operating conditions (performance statistics and retention of multiple devices are shown in Figure S8). This retention performance is comparable to other ferroelectric-based memory devices utilizing vdW ferroelectric heterostructures.^4,32,35^ Furthermore, the on/off ratio remains robust after 10^9^ program/erase cycles (Figure 3d), with only minor V_T_ shifts attributed to bias-stress effects in In_2_O_3_. In addition, the freestanding HZO film itself sustains 10^7^ continuous bipolar switching cycles without polarization degradation (Figure S9), confirming the intrinsic durability of the orthorhombic phase. These results highlight the potential of ferroelectric HZO and ultrathin In_2_O_3_ integration, supported by self-compensating charge balance, as a promising platform for nonvolatile memory technologies.

### Reconfigurable NAND/NOR Based on the Dual-Functional Ferroelectric Inverters

Combining the memory and logic functions of the ferroelectric-dielectric-dual-gated transistors, a reconfigurable multifunction logic device can be developed. We first demonstrate the reconfigurable inverter consisting of a normal load transistor as the pull-up device and a driver transistor based on our dual-gated ferroelectric device, as shown in Figure 4a (optical image in Figure S10). The ferroelectric inverter consists of a connection between two n-type transistors, with the first one single-gated and the second one dual-gated with a ferroelectric HZO top-gate dielectric layer. The first transistor, with its source and local back gate connected, functions as the load, which is similar to a nonlinear resistive pull-up system. The second transistor, which is made of ferroelectric dual-gated device, acts as a driver in enhancement-mode to control the switching of the inverter and modulate the memory state stored in the ferroelectric transistors. The circuit diagram in Figure 4a illustrates the memory operation of the ferroelectric inverter. The driver transistor is made of our dual-gated ferroelectric device, where the top gate V_IN1_ corresponds to the memory gate V_G_^FE^ and the bottom gate V_IN2_ corresponds to the logic gate V_G_^DE^ in the previous sections. Using the property that the ferroelectric polarization states (program/erase states) can be set with a voltage pulse from the top-gate electrode, the V_T_ of the ferroelectric transistors can shift positively or negatively, as shown in Figure 4b. The ″0″ pulse is defined as a 50 ms pulse with V_IN1_ = −2 V, and the ″1″ pulse is defined as a 50 ms pulse with V_IN1_ = +2 V. Figure 4c shows the voltage transfer curves (VTCs) and voltage gain of the ferroelectric inverter device with V_IN1_ ranging from −0.5 to 0.5 V and fixed V_DD_ of 2 V and V_GND_ of 0 V. By varying V_IN1_, the V_T_ of the dual-gate ferroelectric field effect driver can be modulated due to additional control from the top gate. By tuning the V_IN1_ to a positive value, the ultrathin In_2_O_3_ channel accumulates electrons, which causes the I_DS_-V_IN2_ curves to shift toward the negative V_IN2_ region, leading to a negatively shifted VTC. On the contrary, tuning the top gate voltage V_IN1_ to a negative value depletes electrons in the channel, causing the I_DS_-V_IN2_ curves to shift toward the positive V_IN2_ region and leading to a positively shifted VTC.

**Figure 4:**
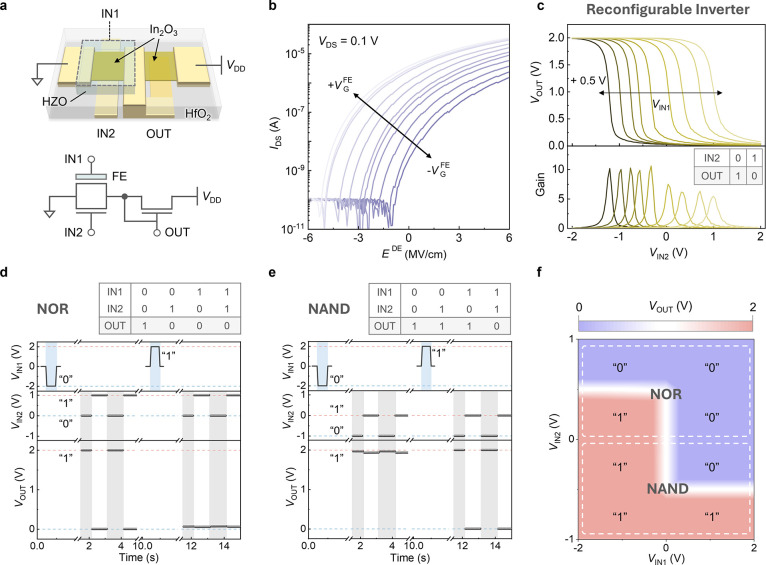
Ferroelectric inverter based on our dual-gate ferroelectric transistors. (a) Schematic of a reconfigurable ferroelectric inverter and the corresponding circuit diagram. (b) Transfer curves of a 2 nm In_2_O_3_ ferroelectric transistor under different V_IN1_. (c) Logic operation of the ferroelectric inverter with different fixed value of V_IN1_, demonstrating programmable V_T_ control. (d) Logic operation and the logic table of the NOR logic based on the ferroelectric inverter. (e) Logic operation and the logic table of the NAND logic based on the ferroelectric inverter. All the output voltages were measured at V_DD_ = 2 V and V_GND_ = 0 V. (f) Reconfigurable dual-gate inverter operation. By redefining the input signals, the same two-transistor NMOS inverter can be dynamically configured to implement either NOR or NAND logic functions.

By utilizing the memory property of the dual-gate ferroelectric driver transistor, additional logic functions can be realized. The demonstration of our reconfigurable NOR/NAND logic gate and the corresponding logic tables is shown in Figure 4d,e. In the NOR/NAND logic gates, the top gate (V_IN1_) and the bottom gate (V_IN2_) in the driver transistor serve as two independent input signals, whereas the output voltage (V_OUT_) is defined as the output signal (with V_OUT_ = 0 V for ″0″ and V_OUT_ = 2 V for ″1″). The input signals are given by a square-wave voltage signal with V_DD_ set at 2 V. For the realization of NOR gate (Figure 4d), IN_1_ = ″0″ (″1″) is defined as an application of a 50 ms pulse with V_IN1_ = −2 V (2 V), and IN_2_ = ″0″ (″1″) is defined as V_IN2_ = 0 V (1 V). When the two inputs are both ″0″ (V_IN1_ = −2 V, V_IN2_ = 0 V), the output is ″1″ (V_OUT_ = 2 V), reflecting the logic properties of the NOR logic gate. For the realization of NAND gate (Figure 4e), IN_1_ = ″0″ (″1″) is defined as an application of a 50 ms pulse with V_IN1_ = −2 V (2 V) the same as the NOR case, and IN_2_ = ″0″ (″1″) is defined as V_IN2_ = −1 V (0 V). When the two inputs are both ″1″ (V_IN1_ = 2 V, V_IN2_ = 0 V), the output is ″0″ (V_OUT_ = 0 V), reflecting the logic properties of the NAND logic gate. Therefore, by simply redefining the input signals, two different logic functions, i.e., NOR and NAND, can be realized in a single inverter circuit with two NMOS transistors, as shown in Figure 4f. Notably, the fabrication of our ferroelectric inverter is fully compatible with BEOL due to the absence of a direct annealing process of HZO thin film on the device, highlighting the potential for future monolithic 3D integration with logic-in-memory applications. Here, BEOL compatibility refers to the entire device-integration flow, including the low process temperature (≤300 °C for ALD In2O3), the employed materials, and the standard lithography, dry-etching, and transfer steps, rather than the thermal budget alone.

### Demonstration of Large Area Transfer of HZO

To meet scalability demands in modern electronics, HZO membranes with lateral dimensions up to approximately 1 cm × 1 cm can be transferred onto bottom-gate transistor substrates, enabling array-level integration. Figure 5a,b show the large-area HZO transfer process and the as-made ultrathin In_2_O_3_-based ferroelectric dual-gated transistors. The HZO thin film remains intact and without noticeable damage after the transfer process. Figure 5c shows the top-gate transfer curves of device arrays based on the large-area transferred HZO with the bottom gate voltage fixed at 0 V. The curves of forward bias sweep (programming of the ferroelectric transistor) are indicated as red lines, whereas those of the backward bias sweep (erasing of the ferroelectric transistor) are indicated as blue lines. The statistical characteristics of the ferroelectric transistors with large area transferred HZO are illustrated in Figure 5d, presenting the forward bias V_T_, backward bias V_T_, and memory window. The average memory window of 1.85 V is achieved, with a standard deviation of 0.11 V and the maximum memory window of 2.03 V. As shown in the statistical distribution of the memory window, the devices with large area transferred HZO retain robust ferroelectricity and good yield throughout the thin film. The transfer and top-gate formation yielded a memory window in 75% of devices (36 out of 48 across two dies; Figure S11), with failures primarily attributed to interface-induced leakage or film cracking issues. The device array exhibits a consistent on-current of 33 μA with a low standard deviation of 8 μA (Figure 5e), confirming the reproducibility of the devices and processes. Figure 5f shows the statistical distribution of the transistor on/off-current ratio and the subthreshold swing (SS) of the large area devices (Figure S12). The devices show high on/off-current ratios and low SS values (60.7 ± 10.2 mV/dec), whereas some devices reached sub-60 mV/dec in the reverse voltage sweeping direction (+V_G_^FE^ to -V_G_^FE^), indicating the effect of ferroelectric polarization switching.^4,12^ From the case of the integration of In_2_O_3_ and large-scale HZO, the large-area transfer of the HZO thin film provides the possibility of fully BEOL fabrication of oxide semiconductor-based ferroelectric transistors and a large array of logic-in-memory systems. The most immediate applications of the present finite-size transferred-HZO/In_2_O_3_ device are BEOL-compatible embedded nonvolatile memory and reconfigurable logic-in-memory blocks that can be integrated at selected regions of a prefabricated CMOS substrate, offering a practical near-term route toward localized 3D heterogeneous integration.

**Figure 5:**
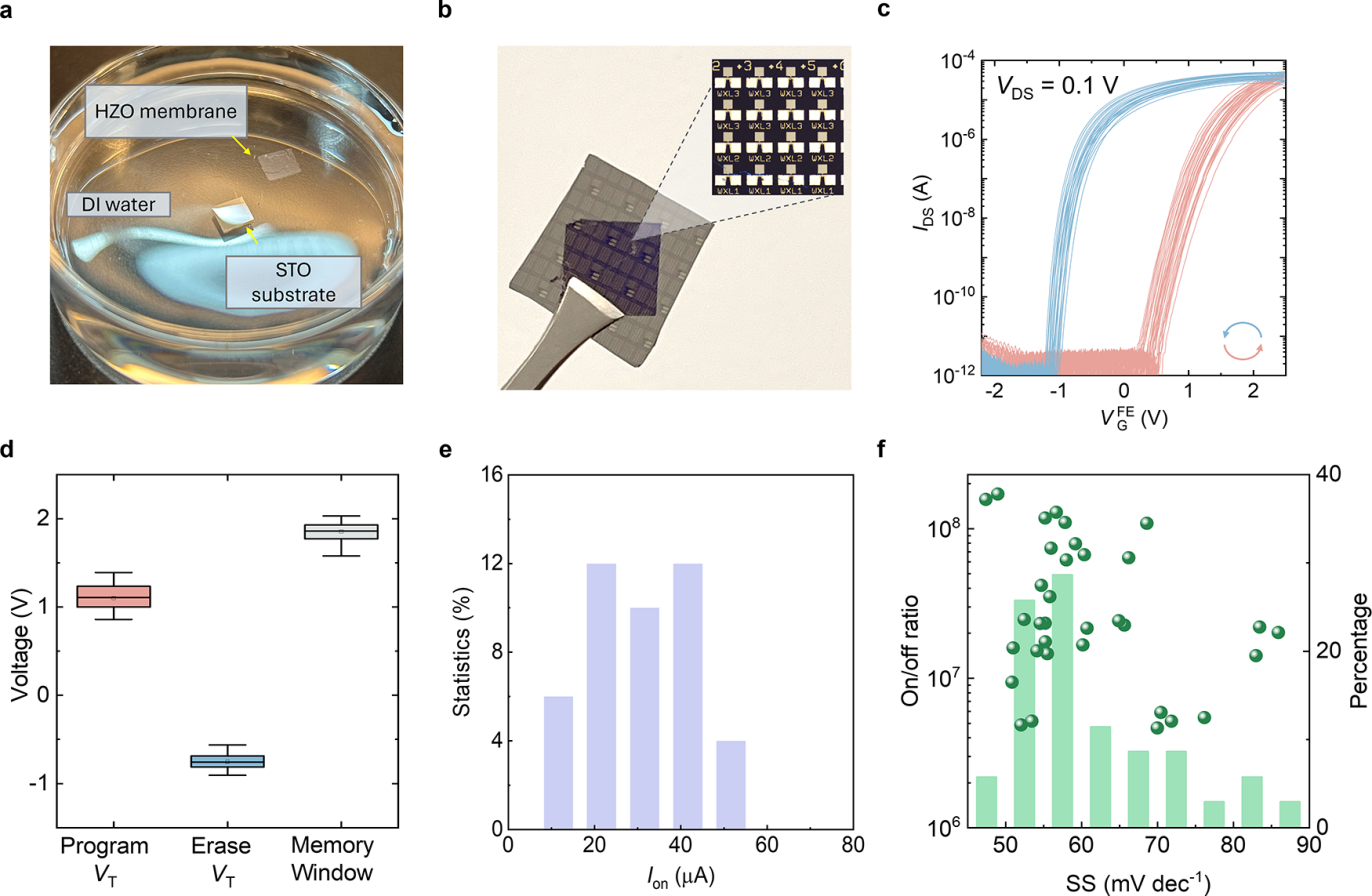
Large area HZO transfer and ferroelectric transistor devices characteristic. (a) Photograph of floating HZO membrane in DI water detached from the STO substrate after wet etching. (b) Optical microscope image of the dual-gate ferroelectric transistor devices fabricated with a large-area transferred 20 nm-thick HZO film. (c) Statistical collection of I_DS_–V_G_^FE^ curves for 2 nm-thick In_2_O_3_ ferroelectric transistors. A stable memory window is shown without significant deviation. (d) Statistical analysis of the program V_T_, erase V_T_, and memory window. The maximum memory window of 2.03 V and the average memory window of 1.85 V is available. (e) Statistical analysis of the on-current for the transistor arrays. (f) Statistical distribution of on/off-current ratios and SS values of large area devices.

## Conclusions

We have developed a transferable HZO membrane process that enables direct, interface-layer-free integration of ferroelectric dielectrics with ultrathin In_2_O_3_ channels, overcoming the interfacial trap formation and polarization screening that limit conventional direct-deposition approaches. By fully crystallizing HZO before transfer, the process preserves the pristine In_2_O_3_ surface and maintains its electrical properties. Furthermore, the unique self-compensation mechanism of In_2_O_3_, where oxygen vacancies and free electrons alternately supply positive and negative charges, ensures complete polarization switching without an interfacial dielectric. The resulting dual-gate ferroelectric transistors exhibit a large and stable memory window, robust endurance, and reduced device-to-device variability, enabling reconfigurable logic-in-memory operation such as dynamic NOR/NAND switching. Fully compatible with back-end-of-line thermal budgets and scalable to large areas, this platform provides a practical route for interface-layer-free ferroelectric logic-in-memory systems.

## Methods

### Growth of HZO and In_2_O_3_

The process begins with the growth of 20 nm-thick HZO thin films on the sacrificial LSMO layer buffered (001)-oriented STO substrate.^4,12^ For the growth of the HZO, pulsed laser deposition (PLD) with a premix KrF (248 nm) excimer laser is used. HZO/La_0.7_Sr_0.3_MnO_3_ (LSMO) heterostructures were grown on (001)-oriented SrTiO_3_ (STO (001)) substrate. To separate HZO thin films and STO substrate, a wet etching method is introduced. The method involves the removal of LSMO sacrificial layers with an acid solution composed of dilute hydrochloric acid (HCl) solution and potassium iodide (KI) as a catalyst. A photoresist layer is spin-coated as the capping layer on top of HZO before immersing it into the etching solution for protection. After the LSMO layer is completely etched, the as-grown HZO with a photoresist capping layer spontaneously lifts off from STO substrate due to the hydrophobicity of photoresist, thereby forming a transferred HZO thin film. Once separated from the STO substrates, the transferred HZO films can be transferred onto arbitrary target substrates/carriers. During the fabrication process of the ferroelectric transistors and inverter, a 2 nm In_2_O_3_ thin film was deposited by atomic layer deposition (ALD) at 275 °C using (CH_3_)_3_In and H_2_O as the precursors.

### Material Characterization

The thickness and the amorphous phase of the as-deposited In_2_O_3_ were measured using a transmission electron microscope (TEM). A focused ion beam (FIB) system was employed to create the cross-sectional specimen, which was subsequently examined using TEM. The θ–2θ X-ray diffraction scans were acquired using a Bruker D2 Phaser at the Core Facility Center, National Cheng Kung University, Taiwan. For polarization-electric field measurements, the Sawyer–Tower circuit was employed.^4,12^ The measurements were conducted using commercial tungsten tips with a peak dimension of approximately 1 μm in diameter. A 20 kHz square wave was applied by a wave generator during the measurement. The capacitance-frequency measurements were recorded by using a commercial impedance/gain-phase analyzer (HP4194A). Using the formula *C* = κ ε_0_ *A*/*d*, the dielectric constant of the transferred HZO could be calculated, where κ is the dielectric constant, ε_0_ = 8.85 × 10^–14^ F cm^–1^ is the vacuum permittivity, *A* is the sample area, and *d* is the thickness of HZO thin films.

### Transistor Fabrication

The device fabrication started with standard cleaning processes of p^+2^ Si substrate with 50 nm SiO_2_. For the part of the bottom gate structure, a 2 nm In_2_O_3_ thin film was deposited by ALD at 275 °C using (CH_3_)_3_In and H_2_O as the precursors. Photolithography and diluted HCl etching were employed to define the In_2_O_3_ active area. Subsequently, 20 nm Ni was deposited using e-beam evaporation, after the standard lithography patterning, followed by a lift-off process to serve as the ohmic contact for the source/drain. For the top-gate structure, the HZO was transferred onto the In_2_O_3_ back-gate structure as the top-gate ferroelectric layer using a wet transfer method. The 20 nm Ni top-gate electrode was then deposited by e-beam evaporation after standard lithography patterning, followed by a lift-off process. After the top-gate formation, an additional photolithography step and a BCl_3_/Ar plasma etching step were carried out to define contact holes through the transferred HZO layer.

### Inverter Fabrication

The inverter devices started with standard cleaning processes for p^+2^ Si substrate with 50 nm SiO_2_. Twenty nm Ni was deposited using e-beam evaporation with bilayer lithography and a lift-off process to serve as the local back gate. Eight nm HfO_2_ was deposited as the gate dielectric by ALD at 250 °C using [(CH_3_)_2_N]_4_Hf (TDMAHf) as the precursor and O_2_ plasma as the oxygen source. A 2 nm In_2_O_3_ thin film was deposited by ALD at 275 °C using (CH_3_)_3_In and H_2_O as the precursors. Lithography and diluted HCl etching were employed to define the In_2_O_3_ active area. The contact hole was patterned using standard lithography, followed by a dry etching process with BCl_3_/Ar plasma. Subsequently, 20 nm Ni was deposited using e-beam evaporation after standard lithography patterning, followed by a lift-off process to serve as the ohmic contact for the S/D and metal line connection. For the part of the top gate structure of the driver transistor, the HZO was transferred into the In_2_O_3_ back gate structure as the top gate ferroelectric layer using a wet transfer method. After transferring the HZO top-gate layer, contact holes were opened by photolithography and BCl_3_/Ar plasma etching prior to deposition of the Ni top-gate electrode, providing probe access to the underlying pads. Finally, a 20 nm Ni top gate electrode of the driver transistor was deposited using e-beam evaporation after standard lithography patterning, followed by a lift-off process.

### Device Characterization

The electrical characteristics of the devices were measured using a Keysight Agilent B2902B source at room temperature, in the absence of light, under atmospheric conditions. Threshold voltage of the transistors was determined by the constant current method defined by *W*/*L*×10^–8^ A. Subthreshold swing (SS value) of the transistors was calculated from the transfer curves by the formula *SS* = *d* V_G_/*d* log­(I_DS_).

## Supplementary Material



## Data Availability

The data that support the findings of this study are available from the corresponding author upon reasonable request.

## Notes

Derivation of the charge-balance relation in the metal–ferroelectric–insulator–semiconductor (MFIS) structure, including a schematic of the Gaussian surface, P–E hysteresis loop of an 8 nm-thick HZO film (Figure S1); TEM–EDX elemental mappings of the transferred-HZO/In2O3 dual-gate ferroelectric transistor (Figure S2); low- and high-magnification TEM characterization of the transferred freestanding HZO film (Figure S3); frequency-dependent C–V measurements of transferred and directly grown HZO films (Figure S4); optical image of the dual-gate ferroelectric transistor (Figure S5); effect of O2 annealing on oxygen-vacancy concentration and memory window (Figure S6); transfer (IDS–VGS) characteristics of the driver transistor in the ferroelectric inverter (Figure S7); retention characteristics of multiple devices (Figure S8); fatigue endurance of the freestanding HZO film (Figure S9); optical image of the ferroelectric inverter (Figure S10); optical image of the large-area transferred membrane and device yield (Figure S11); ferroelectric transistor exhibiting sub-60 mV/decade subthreshold swing (Figure S12); comparison of the memory-window-to-voltage ratio across different works and stack structures (Table S1) (PDF)

## References

[1] Vasić R., Consiglio S., Clark R. D., Tapily K., Sallis S., Chen B., Newby D., Medikonda M., Muthinti G. R., Bersch E., Jordan-Sweet J., Lavoie C., Leusink G. J., Diebold A. C. (2013). Multi-Technique
X-Ray and Optical Characterization of Crystalline Phase, Texture,
and Electronic Structure of Atomic Layer Deposited Hf1–xZrxO2
Gate Dielectrics. J. Appl. Phys..

[2] Lederer M., Kämpfe T., Olivo R., Lehninger D., Mart C., Kirbach S., Ali T., Polakowski P., Roy L., Seidel K. (2019). Local Crystallographic Phase Detection and Texture
Mapping in Ferroelectric Zr-Doped HfO2 Films by Transmission-EBSD. Appl. Phys. Lett..

[3] Cheema S. S., Kwon D., Shanker N., dos Reis R., Hsu S.-L., Xiao J., Zhang H., Wagner R., Datar A., McCarter M. R., Serrao C. R., Yadav A. K., Karbasian G., Hsu C.-H., Tan A. J., Wang L.-C., Thakare V., Zhang X., Mehta A., Karapetrova E. (2020). Enhanced Ferroelectricity in Ultrathin Films
Grown Directly on Silicon. Nature.

[4] Lin C.-Y., Chen B.-C., Liu Y.-C., Kuo S.-F., Tsai H.-C., Chang Y.-M., Kuo C.-Y., Chang C.-F., Chen J.-H., Chu Y.-H., Yamamoto M., Shen C.-H., Chueh Y.-L., Chiu P.-W., Chen Y.-C., Yang J.-C., Lin Y.-F. (2025). Integration
of Freestanding Hafnium Zirconium Oxide Membranes into Two-Dimensional
Transistors as a High-κ Ferroelectric Dielectric. Nat. Electron..

[5] Grimley E. D., Schenk T., Mikolajick T., Schroeder U., LeBeau J. M. (2018). Atomic Structure of Domain and Interphase
Boundaries
in Ferroelectric HfO2. Adv. Mater. Interfaces.

[6] Cho D.-Y., Jung H.-S., Hwang C. S. (2010). Structural Properties and Electronic
Structure of HfO2–ZrO2 Composite Films. Phys. Rev. B.

[7] Mulaosmanovic H., Mikolajick T., Slesazeck S. (2018). Accumulative Polarization Reversal
in Nanoscale Ferroelectric Transistors. ACS
Appl. Mater. Interfaces.

[8] Kim J. Y., Choi M. J., Jang H. W. (2021). Ferroelectric Field Effect Transistors:
Progress and Perspective. APL Mater..

[9] Kim S. J., Mohan J., Summerfelt S. R., Kim J. (2019). Ferroelectric Hf0.5Zr0.5O2
Thin Films: A Review of Recent Advances. JOM.

[10] Park M. H., Kim H. J., Kim Y. J., Lee W., Kim T., Hwang C. S. (2013). Evolution of Phases and Ferroelectric
Properties of
Thin Hf0.5Zr0.5O2 Films According to the Thickness and Annealing Temperature. Appl. Phys. Lett..

[11] Weeks S. L., Pal A., Narasimhan V. K., Littau K. A., Chiang T. (2017). Engineering
of Ferroelectric HfO2–ZrO2 Nanolaminates. ACS Appl. Mater. Interfaces.

[12] Liu Y. C., Chen B. C., Wei C. C., Kuo S. F., Tsai H. C., Chang Y. M., Kuo C. Y., Chang C. F., Chen J. H., Chu Y. H., Yamamoto M., Shen C. H., Chueh Y. L., Chiu P. W., Chen Y. C., Yang J. C., Lin Y. F. (2024). Thickness-Dependent
Ferroelectricity in Freestanding Hf0.5Zr0.5O2Membranes. ACS Appl. Electron. Mater..

[13] Zhong H., Li M., Zhang Q., Yang L., He R., Liu F., Liu Z., Li G., Sun Q., Xie D., Meng F., Li Q., He M., Guo E.-J., Wang C., Zhong Z., Wang X., Gu L., Yang G., Jin K. (2022). Large-Scale Hf0.5Zr0.5O2Membranes with Robust Ferroelectricity. Adv. Mater..

[14] Müller J., Böscke T. S., Schröder U., Mueller S., Bräuhaus D., Böttger U., Frey L., Mikolajick T. (2012). Ferroelectricity
in Simple Binary ZrO2 and HfO2. Nano Lett..

[15] Khan A. I., Keshavarzi A., Datta S. (2020). The Future of Ferroelectric Field-Effect
Transistor Technology. Nat. Electron..

[16] Shibayama S., Nishimura T., Migita S., Toriumi A. (2018). Thermodynamic Control
of Ferroelectric-Phase Formation in HfxZr1-xO2 and ZrO2. J. Appl. Phys..

[17] Huang C., Zhang Y., Zheng S., Yang Q., Liao M. (2021). Interface
Effects Induced by a ZrO2 Seed Layer on the Phase Stability and Orientation
of HfO2 Ferroelectric Thin Films: A First-Principles Study. Phys. Rev. Appl..

[18] Onaya T., Nabatame T., Inoue M., Sawada T., Ota H., Morita Y. (2022). Wake-Up-Free Properties
and High Fatigue Resistance
of HfxZr1–xO2-Based Metal–Ferroelectric–Semiconductor
Using Top ZrO2 Nucleation Layer at Low Thermal Budget (300 °C). APL Mater..

[19] Kim H. J., Park M. H., Kim Y. J., Lee Y. H., Jeon W., Gwon T., Moon T., Kim K. D., Hwang C. S. (2014). Grain Size
Engineering for Ferroelectric Hf0.5Zr0.5O2 Films by an Insertion of
Al2O3 Interlayer. Appl. Phys. Lett..

[20] Onaya T., Nabatame T., Sawamoto N., Ohi A., Ikeda N., Nagata T., Ogura A. (2019). Improvement in Ferroelectricity of
HfxZr1–xO2 Thin Films Using Top- and Bottom-ZrO2 Nucleation
Layers. APL Mater..

[21] Onaya T., Nabatame T., Sawamoto N., Ohi A., Ikeda N., Nagata T., Ogura A. (2017). Improvement in Ferroelectricity of
HfxZr1–xO2 Thin Films Using ZrO2 Seed Layer. Appl. Phys. Express.

[22] Lee S. J., Kim M. J., Lee T. Y., Lee T. I., Bong J. H., Shin S. W., Kim S. H., Hwang W. S., Cho B. J. (2019). Effect
of ZrO2 Interfacial Layer on Forming Ferroelectric HfxZryOz on Si
Substrate. AIP Adv..

[23] Das D., Gaddam V., Jeon S. (2021). Ferroelectricity
in Al2O3/Hf0.5Zr0.5O2
Bilayer Stack: Role of Dielectric Layer Thickness and Annealing Temperature. J. Semicond. Technol. Sci..

[24] Yu E., Lyu X., Si M., Ye P. D., Roy K. (2023). Interfacial
Layer Engineering
in Sub-5-nm HZO: Enabling Low-Temperature Process, Low-Voltage Operation,
and High Robustness. IEEE Trans. Electron Devices.

[25] Si M., Ye P. D. (2021). The Critical Role
of Charge Balance on the Memory Characteristics
of Ferroelectric Field-Effect Transistors. IEEE
Trans. Electron Devices.

[26] Si M., Lyu X., Ye P. D. (2019). Ferroelectric Polarization Switching
of Hafnium Zirconium
Oxide in a Ferroelectric/Dielectric Stack. ACS
Appl. Electron. Mater..

[27] Tasneem N., Kashyap H., Chae K., Park S., Park M. H., Hwang C. S., Jeon S. (2022). Remote Oxygen Scavenging of the Interfacial
Oxide Layer in Ferroelectric Hafnium–Zirconium Oxide-Based
Metal–Oxide–Semiconductor Structures. ACS Appl. Mater. Interfaces.

[28] Hsieh W.-H., Chen Y.-R., Liu Y.-C., Zhao Z., Lee J.-Y., Tu C.-T., Huang B.-W., Wang J.-F., Lee M. H., Liu C. W. (2024). Interfacial-Layer-Free Ge0.95Si0.05 Nanosheet FeFETs. IEEE Trans. Electron Devices.

[29] Shiokawa, T. ; Ichihara, R. ; Hamai, T. Demonstration of High-Performance Ferroelectric HfO2-Based FeFETs. Proc. IEEE Electron Devices Technol. Manuf. Conf. (EDTM) 2023.

[30] Koroleva A. A., Chernikova A. G., Zarubin S. S., Markeev A. M., Zenkevich A. V. (2022). Retention
Improvement of HZO-Based Ferroelectric Capacitors with TiO2 Insets. ACS Omega.

[31] Qi Y., Xu X., Krylov I., Eizenberg M. (2021). Ferroelectricity of As-Deposited
HZO Fabricated by Plasma-Enhanced Atomic Layer Deposition at 300 °C
by Inserting TiO2 Interlayers. Appl. Phys. Lett..

[32] Huang K., Zhai M., Liu X., Sun B., Chang H., Liu J., Feng C., Liu H. (2020). Hf0.5Zr0.5O2 Ferroelectric Embedded
Dual-Gate MoS2 Field Effect Transistors for Memory Merged Logic Applications. IEEE Electron Device Lett..

[33] Si M., Su C. J., Jiang C., Conrad N. J., Zhou H., Maize K., Qiu G., Wu C. T., Ye P. D. (2018). Steep-Slope
Hysteresis-Free Negative Capacitance MoS2 Transistors. Nat. Nanotechnol..

[34] McGuire F. A., Lin Y.-C., Price K., Rayner G. B., Khandelwal S., Salahuddin S., Pop E. (2017). Sustained Sub-60 mV/Decade Switching
via the Negative Capacitance Effect in MoS2 Transistors. Nano Lett..

[35] Xiang J., Chang W. H., Saraya T., Hiramoto T., Irisawa T., Kobayashi M. (2022). Ultrathin
MoS2-Channel FeFET Memory with Enhanced Ferroelectricity
in HfZrO2 and Body-Potential Control. IEEE J.
Electron Devices Soc..

[36] Tseng R., Wang S.-T., Ahmed T., Lin Y.-L., Lee L.-R., Chueh Y.-L., Lin Y.-F. (2023). Wide-Range and Area-Selective
Threshold
Voltage Tunability in Ultrathin Indium Oxide Transistors. Nat. Commun..

[37] Si M., Hu Y., Lin Z., Jiang C., Zhou H., Maize K., Qiu G., Ye P. D. (2021). Why In2O3 Can Make 0.7 nm Atomic Layer Thin Transistors. Nano Lett..

[38] Lin K. K. H., Teng L.-C., Weng T.-T., Lin T.-J., Lin J.-C., Wang S.-Y., Ho P.-H., Woon W.-Y., Kei C.-C., Chou T.-T., Chien C.-H., Lien D.-H. (2024). Suppressing Threshold
Voltage Drift in Sub-2 nm In2O3 Transistors with Improved Thermal
Stability. IEEE Electron Device Lett..

[39] Wang S.-T., Lin Y.-L., Lee L.-R., Ahmed T., Lin Y.-F., Chueh Y.-L. (2024). Reversible Charge
Transfer Doping in Atomically Thin
In2O3 by Viologens. ACS Appl. Mater. Interfaces.

[40] Chang Y.-C., Wang S.-T., Lee Y.-T., Huang C.-S., Hsu C.-H., Weng T.-T., Huang C.-C., Chen C.-W., Chou T.-T., Chang C.-Y., Woon W.-Y., Lin C.-L., Sun J. Y.-C., Lien D.-H. (2025). Breaking the Trade-Off Between Mobility
and On–Off
Ratio in Oxide Transistors. Adv. Mater..

[41] Charnas A., Lin Z., Zhang Z., Ye P. D. (2021). Atomically Thin In2O3 Field-Effect
Transistors with 10̂17 Current On/Off Ratio. Appl. Phys. Lett..

[42] Kirtania, S. G. ; Phadke, O. ; Sarker, E. ; Aabrar, K. A. ; Chakraborty, D. ; Waqar, F. ; Jaewon, S. ; Pantha, T. H. ; Dutta, S. ; Khan, A. ; Yu, S. ; Datta, S. Amorphous Indium Oxide Channel FeFETs with Write Voltage of 0.9 V and Endurance > 10̂12 for Refresh-Free 1T-1FeFET Embedded Memory. Proc. IEEE Int. Electron Devices Meet. (IEDM) 2024, 1–4.

[43] Lin, Z. ; Si, M. ; Ye, P. D. Ultra-Fast Operation of BEOL-Compatible Atomic-Layer-Deposited In2O3 Fe-FETs: Achieving Memory Performance Enhancement with Memory Window of 2.5 V and High Endurance > 10̂9 Cycles without VT Drift Penalty. Proc. IEEE Symp. VLSI Technol. Circuits 2022.

[44] Dutta S., Ye H., Khandker A. A., Kirtania S. G., Khanna A., Ni K., Datta S. (2022). Logic Compatible
High-Performance Ferroelectric Transistor Memory. IEEE Electron Device Lett..

[45] Chen J., Li J., Zhang Q., Xiong S. (2025). High-Performance
Ferroelectric Field-Effect
Transistors Based on Ultrathin Indium Oxide for Neuromorphic Computing. ACS Nano.

[46] Cui T., Chen D., Dong Y., Fan Y., Yao Z., Duan H., Liu J., Liu G., Si M., Li X. (2024). Can Interface Layer Be Really Free for HfxZr1–xO2 Based Ferroelectric
Field-Effect Transistors with Oxide Semiconductor Channel?. IEEE Electron Device Lett..

[47] Li Q., Wang S., Li Z., Zhou H., Wu Y., Qiu G., Maize K., Ye P. D. (2024). High-Performance Ferroelectric Field-Effect
Transistors with Ultra-Thin Indium Tin Oxide Channels for Flexible
and Transparent Electronics. Nat. Commun..

[48] Lu Z., Liu J., Feng J., Zheng X., Yang L. H., Ge C., Jin K. J., Wang Z., Li R. W. (2020). Synthesis of Single-Crystal
La0.67Sr0.33MnO3 Freestanding Films with Different Crystal Orientation. APL Mater..

[49] Li H., Tu J., Du S., Zhang Y., Chen X., Liu Z., Li P., Li X., Wang Y., Gao P. (2025). Efficient Electrodynamic
Stripping for 12-Inch Wafer-Scale Freestanding Ferroelectric Oxide
Membranes. Nat. Commun..

[50] Huang S., Xu S., Ma C., Li P., Guo E.-J., Ge C., Wang C., Xu X., He M., Yang G., Jin K. (2024). Ferroelectric Order Evolution in Freestanding PbTiO3 Films Monitored
by Optical Second Harmonic Generation. Adv.
Sci..

[51] An F., Qu K., Zhong G., Dong Y., Ming W., Zi M., Liu Z., Wang Y., Qi B., Ding Z., Xu J., Luo Z., Gao X., Xie S., Gao P., Li J. (2020). Highly Flexible
and Twistable Freestanding Single Crystalline Magnetite Film with
Robust Magnetism. Adv. Funct. Mater..

[52] Zhao C., Zhao C. Z., Werner M., Taylor S., Chalker P. (2013). Dielectric
Relaxation of High-k Oxides. Nanoscale Res.
Lett..

[53] Dean C. R., Young A. F., Meric I., Lee C., Wang L., Sorgenfrei S., Watanabe K., Taniguchi T., Kim P., Shepard K. L., Hone J. (2010). Boron Nitride Substrates for High-Quality
Graphene Electronics. Nat. Nanotechnol..

[54] Watanabe K., Taniguchi T., Kanda H. (2004). Direct-Bandgap Properties and Evidence
for Ultraviolet Lasing of Hexagonal Boron Nitride Single Crystal. Nat. Mater..

[55] Yang A. J., Han K., Huang K., Lin Y., Li Z., Zhou H., Wu Y., Qiu G., Maize K., Ye P. D. (2022). Van der Waals Integration
of High-κ Perovskite Oxides and Two-Dimensional Semiconductors. Nat. Electron..

[56] Wang X., Zhu C., Deng Y., Qiu G., Zhou H., Wu Y., Maize K., Ye P. D. (2021). Van der
Waals Engineering of Ferroelectric
Heterostructures for Long-Retention Memory. Nat. Commun..

[57] Zhang C., Tu T., Wang J., Liu X., Yang H., Li Z., Zhou H., Wu Y., Qiu G., Maize K., Ye P. D. (2023). Single-Crystalline Van der Waals
Layered Dielectric with High Dielectric
Constant. Nat. Mater..

[58] Huang J. K., Wan Y., Shi J., Xu H., Chen Y., Zhao C., Yang Y., Zhou H., Wu Y., Qiu G., Maize K., Ye P. D. (2022). High-κ Perovskite
Membranes
as Insulators for Two-Dimensional Transistors. Nature.

[59] Ide K., Nomura K., Hosono H., Kamiya T. (2019). Electronic Defects
in Amorphous Oxide Semiconductors: A Review. Phys. Status Solidi A.

[60] Kim M.-K., Kim I.-J., Lee J.-S. (2021). CMOS-Compatible
Ferroelectric NAND
Flash Memory for High-Density, Low-Power, and High-Speed Three-Dimensional
Memory. Sci. Adv..

[61] Si M., Lin Z., Noh J., Li J., Chung W., Ye P. D. (2020). The Impact
of Channel Semiconductor on the Memory Characteristics of Ferroelectric
Field-Effect Transistors. IEEE J. Electron Devices
Soc..

[62] Tseng R., Kuo Y.-H., Pan Y.-Y., Li Z.-H., Wang S.-T., Chen C.-F., Lo S.-T., Chan Y.-C., Wu Y.-J., Chen S.-C., Kuo C.-C., Wang C.-C., Wu C.-H., Lu W.-H., Bao X., Thao N. T. P., Minamitan E., Javey A., Lin C.-L., Lien D.-H. (2026). Dimensional Scaling
Effect in Percolative Oxide Semiconductor Transistors. ACS Nano.

[63] Pan Y.-Y., Hsu C.-H., Tseng R., Wang S.-T., Chang Y.-C., Chen S.-C., Kimura T., Lan Y.-W., Lien D.-H. (2026). Reversible
Thickness Engineering in Amorphous In2O3 Transistors. Nano Lett..

[64] Lin T.-J., Chen S.-C., Lee Y.-T., Cheng S.-L., Tseng R., Wang S.-T., Chang Y.-C., Pan Y.-Y., Chang C.-Y., Chou T.-T., Lin C.-H., Ku C.-S., Lin C.-L., Liu P.-T., Kim H., Lien D.-H. (2026). Origin
of Threshold
Voltage Instabilities in Indium Oxide Transistors. ACS Appl. Mater. Interfaces.

