# Supplementary material for: Reconfigurable Logic-in-Memory Oxide Transistors Enabled by Transferable Ferroelectric HZO
Source: ACS Nano. 2026 Jun 30;20(27):19390–400. doi: 10.1021/acsnano.6c04397 (PMC13374497; doi:10.1021/acsnano.6c04397)
Supplement: Supplementary file 1 [file nn6c04397_si_001.pdf]

Supporting Information

Reconfigurable Logic-in-Memory Oxide Transistors Enabled by Transferable Ferroelectric HZO

Chang-Chang Huang[1‡], Bo-Cia Chen[2,3‡], Hao-Tse Lee[1‡], Rahul[2,3], Yung-Chi Su[4], Chien-Ting Wu[5], Chien-Chung Hsu[5], Yen-Lin Huang[4], Jan-Chi Yang[2,3*], Der-Hsien Lien[1*]

[1] Institute of Electronics, National Yang Ming Chiao Tung University, Hsinchu, Taiwan
[2] Department of Physics, National Cheng Kung University, Tainan, Taiwan
[3] Center for Quantum Frontiers of Research & Technology (QFort), National Cheng Kung University, Tainan, Taiwan
[4] Department of Materials Science and Engineering, National Yang Ming Chiao Tung University, Hsinchu, Taiwan
[5] Taiwan Semiconductor Research Institute (TSRI), Hsinchu, Taiwan

*Address correspondence to: janchiyang@phys.ncku.edu.tw; dhlien@nycu.edu.tw

The derivation of charge balance $Q_s + Q_{de} + Q_{it} = \varepsilon_0\varepsilon_{FE}E_{FE} + P_{FE}$:

The derivation begins from Gauss's law in the differential form,

$$\nabla\cdot\mathbf{E} = \frac{\rho_{tot}}{\varepsilon_0},$$

where $\mathbf{E}$ is the electric field, ε_0 is the vacuum permittivity, and ρ_{tot} denotes the total charge density, including both free and bound charges. The total charge density can be decomposed as

$$\rho_{tot} = \rho_f + \rho_b$$

where ρ_f is the free charge density and ρ_b is the bound charge density arising from material polarization. The bound charge density is related to the polarization vector $\mathbf{P}$ by

$$\rho_b = -\nabla\cdot\mathbf{P}$$

where $\mathbf{P}$ denotes the polarization density. Substituting this relation into Gauss's law leads to

$$\nabla\cdot(\varepsilon_0\mathbf{E}) = \rho_f - \nabla\cdot\mathbf{P}$$

which can be rearranged as

$$\nabla\cdot(\varepsilon_0\mathbf{E} + \mathbf{P}) = \rho_f$$

This naturally motivates the definition of the electric displacement field,

$$\mathbf{D} \equiv \varepsilon_0\mathbf{E} + \mathbf{P}$$

such that Gauss's law takes the compact form

$$\nabla\cdot\mathbf{D} = \rho_f$$

To apply this relation to the MFIS structure, a rectangular Gaussian surface is constructed along the z direction. The upper face of the Gaussian surface lies within the ferroelectric layer, while the lower face is positioned just outside the semiconductor region, immediately adjacent to the semiconductor interface. Under this construction, the electric field and polarization vanish at infinity, such that the displacement flux through the far-end face is zero. Furthermore, under the one-dimensional approximation, the electric field and polarization are assumed to be uniform and normal to the interfaces, so that the sidewall contribution to the flux also vanishes. As a result, the surface integral of $\mathbf{D}$ reduces to the contribution from the single face located inside the ferroelectric layer.

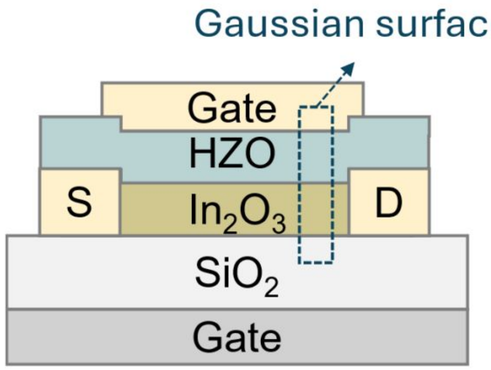

Schematic of Gaussian surface in MFIS stack

Integrating Gauss's law over this Gaussian volume gives

$$\oint \mathbf{D} \cdot d\mathbf{S} = Q_f$$

where Q_f is the total free charge enclosed by the Gaussian surface.

Dividing both sides by the cross-sectional area A of the Gaussian surface, the relation can be expressed in terms of free charge density,

$$D_{\mathrm{FE}} = \sigma_f.$$

Here, σ_f represents the net free charge density required to terminate the displacement field emerging from the ferroelectric layer. In the MFIS structure, this free charge density is provided by the semiconductor channel charge density Q_s, the free charge density in the dielectric layer Q_{de}, and the interface trap charge density Q_{it} associated with trapped and fixed charges at the interfaces, all defined per unit area, such that

$$D_{\mathrm{FE}} = Q_s + Q_{\mathrm{de}} + Q_{\mathrm{it}}.$$

The displacement field inside the ferroelectric layer can be written as

$$D_{\mathrm{FE}} = \varepsilon_0 E_{\mathrm{FE}} + P_{\mathrm{tot}},$$

where E_{FE} is the electric field in the ferroelectric layer and P_{tot} denotes the total polarization. The total polarization is decomposed into a switchable ferroelectric polarization and a linear dielectric contribution,

$$P_{\mathrm{tot}} = P_{\mathrm{FE}} + P_{\mathrm{lin}},$$

with the linear polarization given by

$$P_{\mathrm{lin}} = \varepsilon_0 \chi_e E_{\mathrm{FE}},$$

where χ_e is the electric susceptibility of the linear polarization in the ferroelectric layer. Substituting this expression yields

$$D_{\mathrm{FE}} = \varepsilon_0 (1 + \chi_e) E_{\mathrm{FE}} + P_{\mathrm{FE}}.$$

Using the relation $\varepsilon_{\mathrm{FE}} = 1 + \chi_e$, we finally obtain

$$Q_s + Q_{\mathrm{de}} + Q_{\mathrm{it}} = \varepsilon_0 \varepsilon_{\mathrm{FE}} E_{\mathrm{FE}} + P_{\mathrm{FE}}.$$

In this expression, Q_s, Q_{de}, and Q_{it} are defined as charge densities per unit area. Q_s originates from the semiconductor channel and Q_{de} from the non-ferroelectric free charge on the dielectric side, while Q_{it} denotes the total interface trap charge, including both the top HZO/In_2O_3 interface and the substrate-side SiO_2/In_2O_3 interface. The ferroelectric bound charge associated with polarization is fully accounted for by P_{FE} and is therefore not included in Q_s, Q_{de}, or Q_{it}.

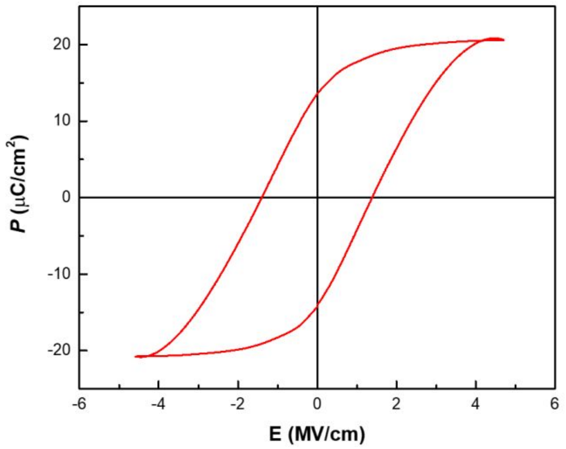

Figure S1. Polarization–electric field (*P*–*E*) hysteresis loop of an 8 nm-thick HZO film. The thinner film exhibits a higher remanent polarization than the 20 nm film used in this work, consistent with the thickness-dependent ferroelectricity of freestanding HZO.

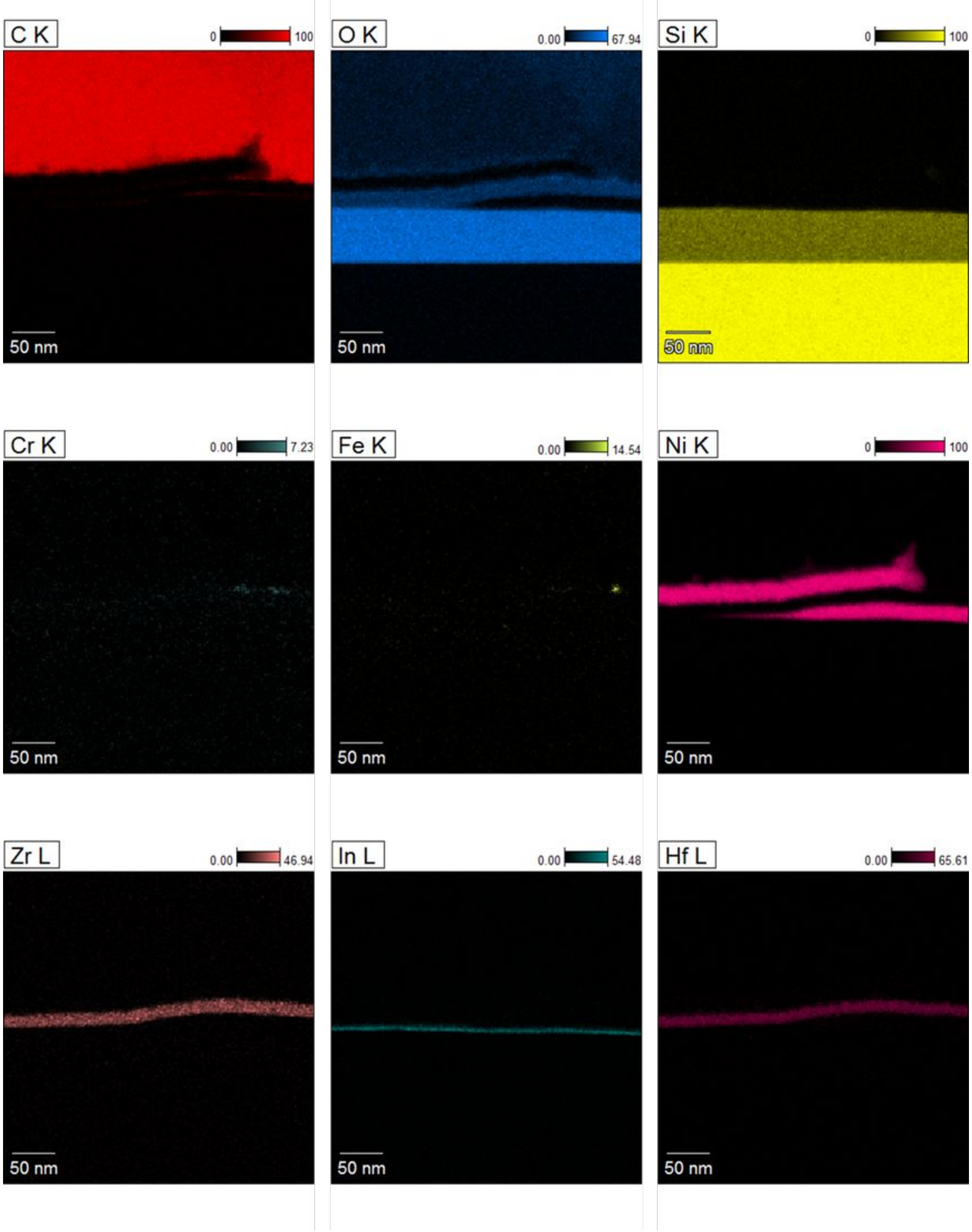

Figure S2. The TEM-EDX mappings. Zoomed-in EDX elemental mapping of the dual-gated ferroelectric transistor based on a transferable ferroelectric HZO membrane of 20 nm thickness and ultrathin In_2O_3 of 2 nm thickness. The scale bars are 50 nm.

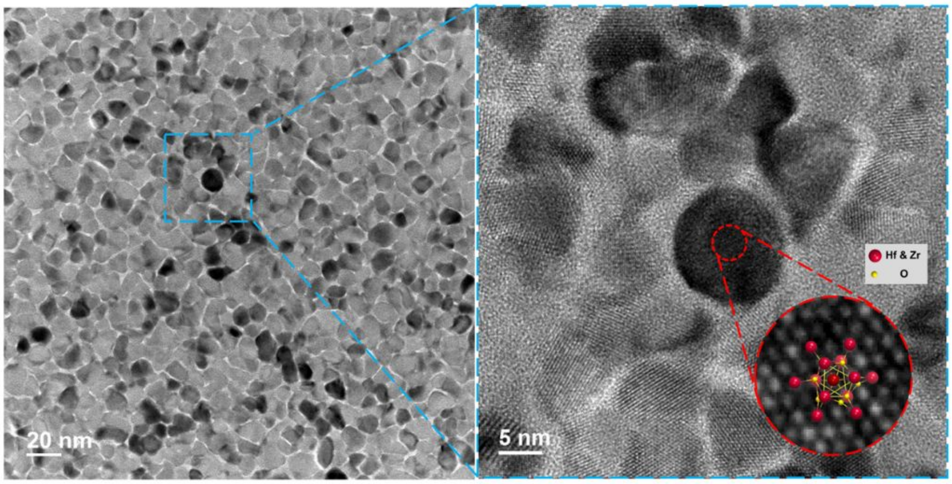

Figure S3. Low- and high-magnification TEM characterizations of the transferred FS-HZO film. The low-magnification TEM image reveals a dense polycrystalline microstructure composed of ultrafine nanoscale grains distributed throughout the transferred membrane. The red-circled region highlights the corresponding atomic-resolution image and the local atomic configuration illustration associated with the orthorhombic ferroelectric phase of HZO.

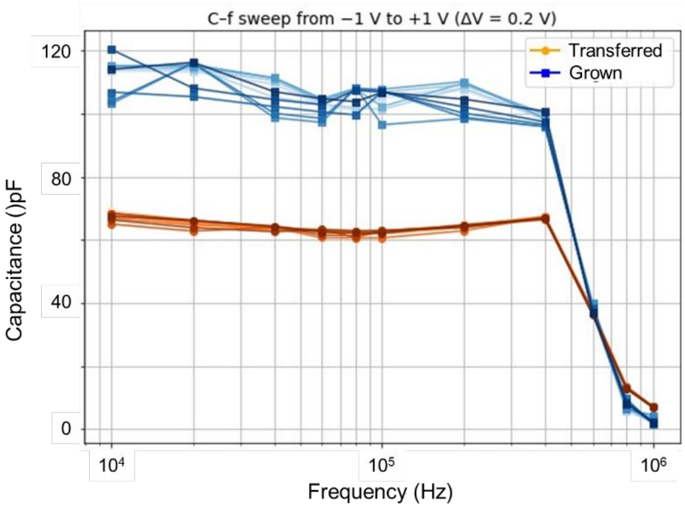

Figure S4. Frequency-dependent C-V measurements for transferred HZO and directly grown HZO films from 10 kHz to 1 MHz, under a bias sweeping from -1 V to +1 V.

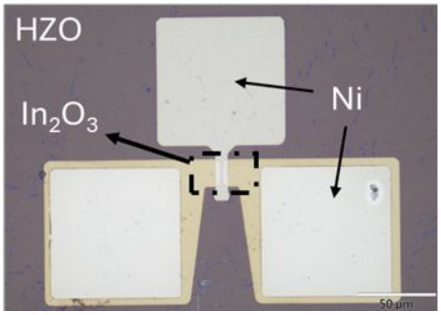

Figure S5. Optical image of the dual-gated ferroelectric transistor based on transferable ferroelectric HZO and ultrathin In_2O_3.

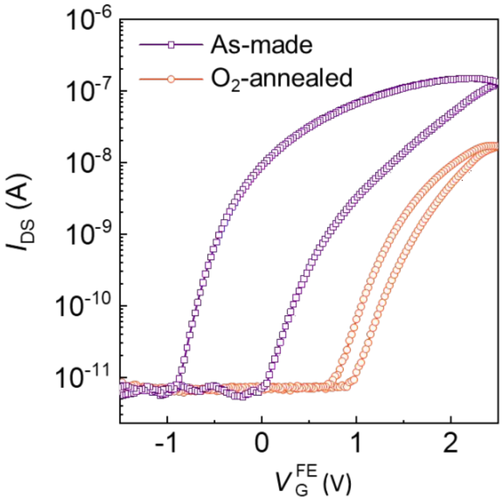

Figure S6. After O_2 annealing at 150 ^{o}C for 1 hour, the oxygen vacancy concentration could be reduced, which leads to a reduction in the memory window.

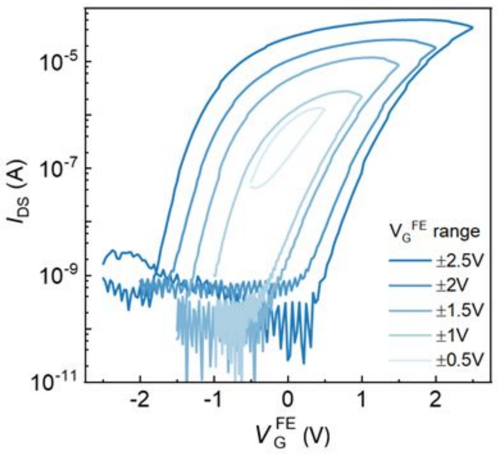

Figure S7. I_{DS}–V_G^{FE} curve for the driver transistor in our ferroelectric inverter device. Stable counterclockwise hysteresis loops with different ferroelectric-gate voltage ranges are available. The device has a channel width of 30 μm and a channel length of 2 μm, with a drain-to-source voltage V_{DS} = 0.1 V.

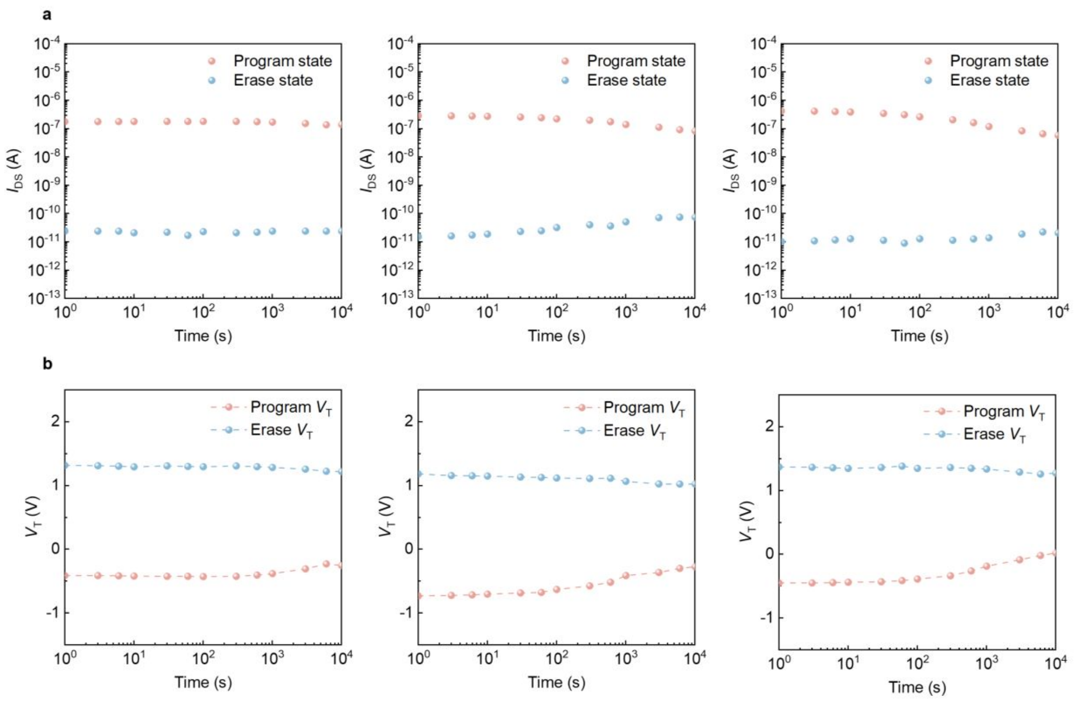

Figure S8. Retention characterization of multiple devices. (a) I_{DS} evolution in the program and erase states as a function of time. All measurements were performed at V_{DS} = 0.1 V, with the drain current measured at $V_G{}^{\mathrm{FE}}$ = 0.5 V and $V_G{}^{\mathrm{DE}}$ = 0 V. (b) Corresponding threshold voltage evolution during retention. The initial program/erase pulse amplitude is +3 V/-3 V with a pulse width of 50 ms. A total of 36 devices were characterized under identical measurement conditions. All electrical measurements were performed at V_{DS} = 0.1 V, with $V_G{}^{\mathrm{FE}}$ swept from $-$1.5 V to +1.5 V.

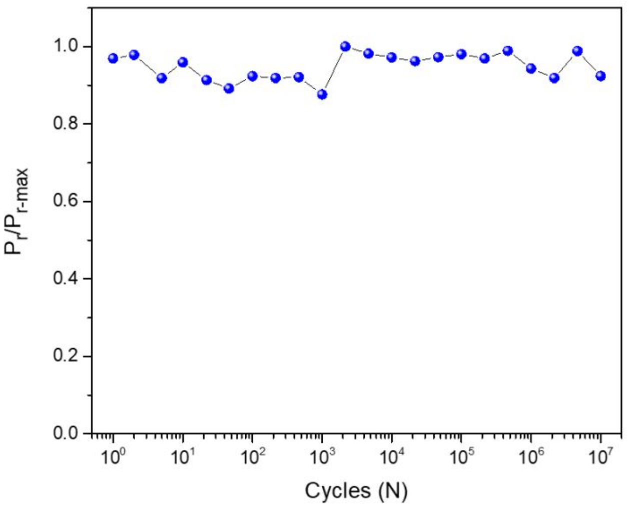

Figure S9. Fatigue endurance characteristics of the freestanding HZO film measured on the LSMO/STO substrate prior to device integration. The normalized remanent polarization ($P/P_{r,max}$) remains highly stable without noticeable degradation up to 10^7 bipolar switching cycles, indicating that the freestanding HZO layer preserves robust ferroelectric durability after the transfer-related processing.

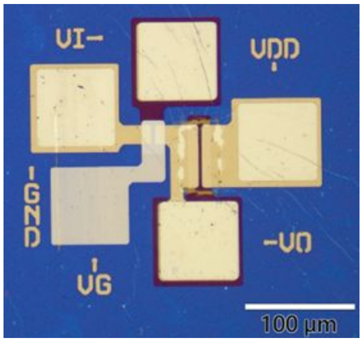

Figure S10. Optical image of the ferroelectric inverter based on our dual-gated ferroelectric transistor with transferable ferroelectric HZO and ultrathin In_2O_3.

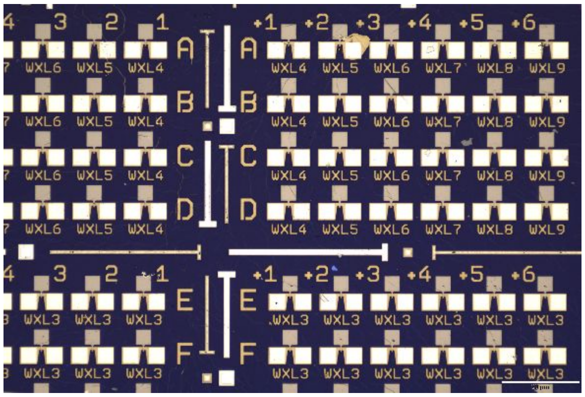

Figure S11. Optical image of the transferred membrane. The yield of In_2O_3 devices exhibiting a memory window is approximately 75% (36 out of 48 total devices across two dies).

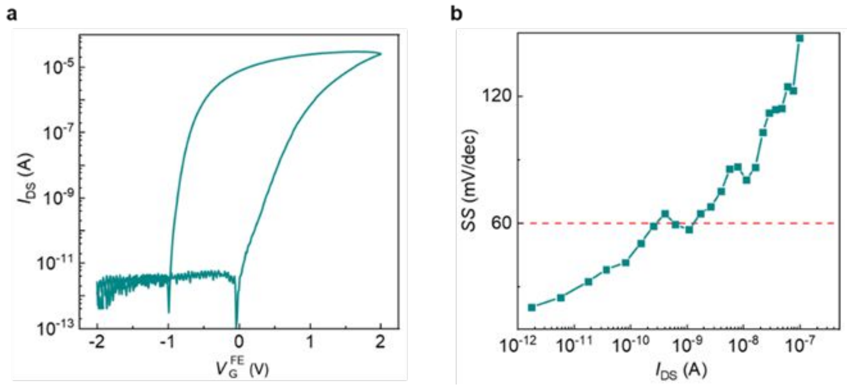

Figure S12. A ferroelectric transistor device showcasing sub-60 mV/decade subthreshold swing. (a) I_{DS}–$V_G{}^{FE}$ curve for the 2 nm-thick In_2O_3 ferroelectric transistor. A stable counterclockwise hysteresis is available. (b) The extracted subthreshold swing of the 2 nm-thick In_2O_3 ferroelectric transistor device. SS-values below 60 mV/decade are achieved. The device has a channel width of 10 μm and a channel length of 3 μm, with a drain-to-source voltage V_{DS} = 0.1 V.

Stack structure	Interfacial layer	Structure	Memory window (V)	Sweeping voltage (V)	MW-$V_{G,max}^{FE}$ ratio	Reference
Transferable HZO/MoS_2	No	Top-gate	0.05	4	0.0125	1
HZO superlattice/$Ge_{0.95}Si_{0.05}$	No	Nanosheet	1.8	4	0.45	2
HfO_2/TiO_2	No	Bottom-gate	1.3	8	0.1625	3
HZO/MoS_2	No	Bottom-gate	1	12	0.083	4
HZO/Al_2O_3/MoS_2	Yes	Bottom-gate	0.012	2	0.006	5
TiN/HZO/TiN/HfO_2/MoS_2	Yes	Bottom-gate	6	16	0.375	6
HZO/ZrO_2/MoS_2	Yes	Bottom-gate	0.22	2.3	0.0956	7
HZO/IWO	No	Bottom-gate	1	2	0.5	8
HZO/Al_2O_3/In_2O_3	Yes	Bottom-gate	2.5	8	0.3125	9
HZO/IWO	No	Bottom-gate	1.6	4	0.4	10
HZO/IGZO	No	Bottom-gate	0	6	0	11
HZO/ITO	No	Bottom-gate	2.78	10	0.278	12
HZO/IZO	No	Bottom-gate	2	10	0.2	13
HZO/GaN	No	Top-gate	0.11	12	0.009	14
HZO/Ge	No	Top-gate	4.8	9	0.53	14
Transferable HZO/In_2O_3	No	Top-gate	2.3	2.5	0.48	This work

Table S1. Comparison of MW-$V_{G,max}{}^{FE}$ ratio in different works and stack structures.

References

(1) Lin, C.-Y.; Chen, B.-C.; Liu, Y.-C.; Kuo, S.-F.; Tsai, H.-C.; Chang, Y.-M.; Kuo, C.-Y.; Chang, C.-F.; Chen, J.-H.; Chu, Y.-H.; Yamamoto, M.; Shen, C.-H.; Chueh, Y.-L.; Chiu, P.-W.; Chen, Y.-C.; Yang, J.-C.; Lin, Y.-F. Integration of Freestanding Hafnium Zirconium Oxide Membranes into Two-Dimensional Transistors as a High-κ Ferroelectric Dielectric. *Nat. Electron.* **2025**, *8*, 560–570.

(2) Hsieh, W.-H.; Chen, Y.-R.; Liu, Y.-C.; Zhao, Z.; Lee, J.-Y.; Tu, C.-T.; Huang, B.-W.; Wang, J.-F.; Lee, M. H.; Liu, C. W. Interfacial-Layer-Free Ge0.95Si0.05 Nanosheet FeFETs. *IEEE Trans. Electron Devices* **2024**, *71*, 1758–1763.

(3) Shiokawa, T.; Ichihara, R.; Hamai, T.; et al. Demonstration of High-Performance Ferroelectric HfO2-Based FeFETs. *Proc. IEEE Electron Devices Technol. Manuf. Conf. (EDTM)* **2023**.

(4) Huang, K.; Zhai, M.; Liu, X.; Sun, B.; Chang, H.; Liu, J.; Feng, C.; Liu, H. Hf0.5Zr0.5O2 Ferroelectric Embedded Dual-Gate MoS2 Field Effect Transistors for Memory Merged Logic Applications. *IEEE Electron Device Lett.* **2020**, *41*, 1600–1603.

(5) Si, M.; Su, C. J.; Jiang, C.; Conrad, N. J.; Zhou, H.; Maize, K.; Qiu, G.; Wu, C. T.; Ye, P. D. Steep-Slope Hysteresis-Free Negative Capacitance MoS2 Transistors. *Nat. Nanotechnol.* **2018**, *13*, 24–28.

(6) McGuire, F. A.; Lin, Y.-C.; Price, K.; Rayner, G. B.; Khandelwal, S.; Salahuddin, S.; Pop, E. Sustained Sub-60 mV/Decade Switching via the Negative Capacitance Effect in MoS2 Transistors. *Nano Lett.* **2017**, *17*, 4801–4806.

(7) Xiang, J.; Chang, W. H.; Saraya, T.; Hiramoto, T.; Irisawa, T.; Kobayashi, M. Ultrathin MoS2-Channel FeFET Memory with Enhanced Ferroelectricity in HfZrO2 and Body-Potential Control. *IEEE J. Electron Devices Soc.* **2022**, *10*, 72–77.

(8) Kirtania, S. G.; Phadke, O.; Sarker, E.; Aabrar, K. A.; Chakraborty, D.; Waqar, F.; Jaewon, S.; Pantha, T. H.; Dutta, S.; Khan, A.; Yu, S.; Datta, S. Amorphous Indium Oxide Channel FeFETs with Write Voltage of 0.9 V and Endurance >1012 for Refresh-Free 1T-1FeFET Embedded Memory. *Proc. IEEE Int. Electron Devices Meet. (IEDM)* **2024**, 1–4.

(9) Lin, Z.; Si, M.; Ye, P. D. Ultra-Fast Operation of BEOL-Compatible Atomic-Layer-Deposited In2O3 Fe-FETs: Achieving Memory Performance Enhancement with Memory Window of 2.5 V and High Endurance >109 Cycles without VT Drift Penalty. *Proc. IEEE Symp. VLSI Technol. Circuits* **2022**.

(10) Dutta, S.; Ye, H.; Khandker, A. A.; Kirtania, S. G.; Khanna, A.; Ni, K.; Datta, S. Logic Compatible High-Performance Ferroelectric Transistor Memory. *IEEE Electron Device Lett.* **2022**, *43*, 382–385.

(11) Cui, T.; Chen, D.; Dong, Y.; Fan, Y.; Yao, Z.; Duan, H.; Liu, J.; Liu, G.; Si, M.; Li, X. Can Interface Layer Be Really Free for HfxZr1 − xO2 Based Ferroelectric Field-Effect Transistors with Oxide Semiconductor Channel? *IEEE Electron Device Lett.* **2024**, *45*, 368–371.

(12) Li, Q.; Wang, S.; Li, Z.; Zhou, H.; Wu, Y.; Qiu, G.; Maize, K.; Ye, P. D. High-Performance Ferroelectric Field-Effect Transistors with Ultra-Thin Indium Tin Oxide Channels for Flexible and Transparent Electronics. *Nat. Commun.* **2024**, *15*, 2686.

(13) Kim, M.-K.; Kim, I.-J.; Lee, J.-S. CMOS-Compatible Ferroelectric NAND Flash Memory for High-Density, Low-Power, and High-Speed Three-Dimensional Memory. *Sci. Adv.* **2021**, *7*, eabe1341.

(14) Si, M.; Lin, Z.; Noh, J.; Li, J.; Chung, W.; Ye, P. D. The Impact of Channel Semiconductor on the Memory Characteristics of Ferroelectric Field-Effect Transistors. *IEEE J. Electron Devices Soc.* **2020**, *8*, 846–849.
